# Design of Variable Spray System for Plant Protection UAV Based on CFD Simulation and Regression Analysis

**DOI:** 10.3390/s21020638

**Published:** 2021-01-18

**Authors:** Ming Ni, Hongjie Wang, Xudong Liu, Yilin Liao, Lin Fu, Qianqian Wu, Jiong Mu, Xiaoyan Chen, Jun Li

**Affiliations:** 1 College of Information Engineering, Sichuan Agricultural University, Ya’an 625000, China; nm@sicau.edu.cn (M.N.); kobosp@stu.sicau.edu.cn (H.W.); liaoyilin2000@163.com (Y.L.); F32056128@163.com (L.F.); qian1092020@163.com (Q.W.); jmu@sicau.edu.cn (J.M.); chenxy@sicau.edu.cn (X.C.); 2 Sichuan Key Laboratory of Agricultural Information Engineering, Ya’an 625000, China; 3 College of Science, Sichuan Agricultural University, Ya’an 625000, China; tianwen_2002@163.com

**Keywords:** aviation plant protection, downwash wind field, deposition distribution characteristic, support vector regression, back propagation neural network, farmland experiment

## Abstract

Multi-rotor unmanned aerial vehicles (UAVs) for plant protection are widely used in China’s agricultural production. However, spray droplets often drift and distribute nonuniformly, thereby harming its utilization and the environment. A variable spray system is designed, discussed, and verified to solve this problem. The distribution characteristics of droplet deposition under different spray states (flight state, environment state, nozzle state) are obtained through computational fluid dynamics simulation. In the verification experiment, the wind velocity error of most sample points is less than 1 m/s, and the deposition ratio error is less than 10%, indicating that the simulation is reliable. A simulation data set is used to train support vector regression and back propagation neural network with multiple parameters. An optimal regression model with the root mean square error of 6.5% is selected. The UAV offset and nozzle flow of the variable spray system can be obtained in accordance with the current spray state by multi-sensor fusion and the predicted deposition distribution characteristics. The farmland experiment shows that the deposition volume error between the prediction and experiment is within 30%, thereby proving the effectiveness of the system. This article provides a reference for the improvement of UAV intelligent spray system.

## 1. Introduction

China has a large population, thus ensuring food security is of great importance to national security and people’s life [1]. However, grain production is considerably reduced due to the influence of diseases, pests, grasses, and animals. From 2009 to 2018, the grain loss recovered by taking control measures accounted for 15.6% of the total grain output [2]. However, the pesticide utilization rate of China in 2019 is only 39.8% [3]. The abuse of pesticides affects the food safety and easily causes environment pollution [4]. The documents issued by the State Council of China clearly require to achieve reduction in pesticide usage, strengthen the research and development of intelligent agricultural equipment, and realize agricultural mechanization in China [5].

Precision agriculture (PA) is a concept and trend of agricultural modernization and can be expressed as applying the right practice at the right place, at the right time, and with the right quantity [6]. As the representative of precision agriculture, aviation plant protection technology has been continuously applied and improved in the fields of farmland remote sensing, disease and pest identification, and intelligent spraying [7,8]. Large-scale heavy-duty plant protection aircraft have the characteristics of heavy load, long range, and wide coverage, but they are gradually being replaced by small drones in the development of precision agriculture in China [9]. Plant protection unmanned aerial vehicle (UAV) has low application cost (education, purchase, and maintenance), strong mobility, free from site constraints, close distance to the crops, and can effectively reduce the harm to operators during pesticide application [10,11]. Since 2014, plant protection UAV has shown a rapid development trend [12]. Although plant protection UAV has many advantages, the droplet deposition is easy to be affected by multiple factors, resulting in repeated spray, missing spray, and less spray [13,14]. This condition reduces the utilization rate of pesticides and affects the ecological balance of the region.

Many studies have been conducted on the effect factors of spray droplet deposition in plant protection UV, as shown in Table 1. The droplet diameter is the volume median diameter (VMD), and the volume of droplets smaller than this diameter accounts for 50% of the total volume of droplets [15]. However, these studies cannot fully show the effect of multiple factors on the deposition distribution characteristics due to some limitations. In addition to these traditional factors, Guo et al. found that the vortex size directly affects the result of spray operation [16]. Cheng et al. studied droplet deposition distribution uniformity under different canopy densities [17]. Measuring the vortex size and canopy density quantitatively is difficult. However, numerical simulation can conveniently consider most of the factors affecting the deposition distribution characteristics.

Computational fluid dynamics (CFD) is the product of the combination of modern fluid mechanics, numerical mathematics, and computer science. It has been widely used in industrial design and agricultural plant protection [29]. Lattice Boltzmann method (LBM) and finite volume method (FVM) are two branches of CFD algorithm. The former is dedicated to solving the Navier–STokes equation of fluid, and the latter is to simulate the whole fluid motion by calculating the streaming and collisional processes between microscopic particles [29]. Tang et al. used LBM to effectively simulate the unsteady flow caused by the main rotor of plant protection helicopter [30]. However, LBM often leads to a large amount of calculation, high equipment requirement, and low resolution [29]. Thus, mature FVM is often used to simulate the wind field of plant protection UAV for analyzing the effect of different flight and environment states [31,32,33,34]. The result shows that this method is reliable by using an anemometer or particle image velocimetry [35]. For droplet deposition, Zhang et al. designed a near ground platform to precisely simulate aerial spray and optimize spray parameters [36]. However, the platform cost is high, and the vertical movement range is limited by the floor height. The range of CFD simulation parameters is not limited by the environment; thus, they are widely used in the evaluation of droplet deposition distribution characteristics [37,38,39]. Hong et al. used CFD to simulate pesticide spray from air-assisted sprays in an apple orchard. The overall relative errors of the spray concentration inside canopy losses are 22.1%, thereby showing that CFD is reliable even in complex environment [40]. Therefore, compared with field and wind tunnel experiments, CFD is an efficient, reliable, low-cost method to obtain a large number of droplet deposition distribution characteristics in the case of multiple factors.

Regression analysis is a set of statistical processes used to estimate the relationships between a dependent variable and one or more independent variables [41]. With the development of machine learning and the improvement of hardware, regression analysis emerges in many agricultural scenarios. In the spray effect evaluation, the regression model is trained using the simulation data under finite state, and the spray effect under unknown state is predicted by the model [42,43]. Yang et al. analyzed the effect of multiple factors on droplet drift by combining CFD data verified by experiments and radial basis function regression model [44]. The results show that regression analysis can fit the simulation data well and reduce the cost of experiment and modeling. Guo et al. used multiple regression models to predict the spray effect under twin nozzles [45]. Huang et al. used a multivariate linear regression model to predict the deposition of chemical solution after operation [46]. The experimental results show that the regression models with simple architecture are feasible, but are limited to the case without propeller and should be improved for big and nonlinear data. Wang et al. used partial least squares regression and support vector machine (SVM) to assess the nitrogen status of tea plants [47]. Campos et al. used a decision support system to generate the dosage of UAV spray in vineyards [48]. Wen et al. used artificial neural networks (ANNs) to predict droplet deposition [49]. They all achieved good results, thereby showing that the fitting ability of regression model varies with different agricultural scenarios [50]. Previous works illustrated the operability of a variable spray system based on CFD simulation and regression analysis.

Plant protection UAV with variable spray system is the product of hardware and software integration. Wen et al. designed a precision variable spray system based on single chip microcomputer and micro diaphragm pump. This system can control the pump flow in real time in accordance with proportional-integral-differential control and pulse width modulation, thereby proving that the variable spray system is feasible in hardware [51,52]. At the software level, the main reference factor is the standard for determining the dosage. Hong et al. achieved a variable spray system for rice canopy coverage [53], Hunter et al. obtained the weedy area [54], and Wang et al. aimed to avoid planting areas without crops [55]. Wen et al. designed a variable spray system based on wind tunnel test data and neural network model [49]. The outdoor experimental results show that this system can spray in accordance with the demand in complex states. The spray system of Liu et al. is uniform spray, and the control mode is empirical mathematical model [56]. This system has high stability, precision, and automation. However, the variable spray system described above mainly controls the nozzle flow and does not consider the droplet drift. The experimental results of Wang et al. show that the droplet drift has a nonnegligible role on the spray effect [57], but changing the UAV type or adding adjuvants is inconvenient. Therefore, a variable spray system that can adjust the nozzle flow and solve the problem of droplet drift is important.

This study attempts to establish the relationship among UAV flight state (propeller speed, pitch angle, flight height), environment state (wind velocity, environment temperature, environment humidity), spray state (nozzle flow, droplet diameter, distance between propeller and nozzle), and spray droplet deposition distribution characteristics (effective deposition radius, center offset distance). A variable spray system with adjustable nozzle flow and controllable UAV offset is designed. In this study, the CFD simulation provides the data source, and the field experiment verifies the correctness of the data. Multiple regression methods are used to establish the relationship between sensor states and distribution characteristics. The effectiveness of a multi-sensor fusion variable spray system is verified through real farmland spray.

The rest of this paper is organized as follows. Section 2 introduces and verifies the effectiveness of the FVM and discrete phase model (DPM). Section 3 analyzes the spray droplet deposition distribution characteristic under different states. Section 4 introduces and compares various regression models to select the best one for predicting the distribution characteristics. Section 5 introduces the design, experiment, and evaluation of variable spray system. Section 6 provides the conclusion and future work.

## 2. Application and Verification of CFD Simulation

### 2.1. FVM and Turbulence Model

Compared with aerospace wind tunnel, plant protection UAV wind tunnel is a low-velocity wind tunnel. Therefore, the wind field of UAV can be directly considered a 3D, steady, isothermal, incompressible, viscous, and turbulent flow. Relevant studies show that the shear-stress transport (SST) *k*–ω turbulence model can accurately capture the flow separation characteristics, simulate the turbulence phenomenon realistically, and calculate the aerodynamic parameters accurately. This model is widely used in the numerical simulation of propeller wind field [32,37,58]. The CFD simulation in this paper is based on Fluent (2019 R2, ANSYS Inc., Canonsburg, USA) simulation platform.

Fluid flow is governed by conservation laws. The basic conservation laws include mass conservation, momentum conservation, and energy conservation. The governing equation shown in Formula (1) is the mathematical expression of these conservation laws.

(1) $$\begin{array}{ccc} \frac{\partial \rho }{\partial t}+\frac{\partial (\rho u)}{\partial x}+\frac{\partial (\rho v)}{\partial y}+\frac{\partial (\rho w)}{\partial z} & = & 0,\\ \frac{\partial (\rho u)}{\partial t}+div\left(\rho u\vec{u}\right) & = & div\left(\mu \;grad\;u\right)-\frac{\partial p}{\partial x}+S_{u},\\ \frac{\partial (\rho v)}{\partial t}+div\left(\rho v\vec{u}\right) & = & div\left(\mu \;grad\;v\right)-\frac{\partial p}{\partial y}+S_{v},\\ \frac{\partial (\rho w)}{\partial t}+div\left(\rho w\vec{u}\right) & = & div\left(\mu \;grad\;w\right)-\frac{\partial p}{\partial z}+S_{w},\\ \frac{\partial (\rho T)}{\partial t}+div\left(\rho T\vec{u}\right) & = & div\left(\frac{k}{C_{p}}\;grad\;T\right)+S_{T}. \end{array}$$

In Formula (1), ρ is the density, *t* is the time, *p* is the pressure, and $\vec{u}$ is the velocity vector. *u*, *v*, *w* are the components of $\vec{u}$ in the three directions of X, Y, Z. μ is the dynamic viscosity, and $S_{u}$, $S_{v}$, $S_{w}$ are the generalized source terms of the momentum equation. *T* is the specific heat capacity, *k* is the heat conduction coefficient, $C_{p}$ is the specific heat capacity, and $S_{T}$ is the heat dissipation of fluid mechanical energy.

In Formula (2), the *k*–ω turbulence model is used for numerical simulation. $\mu_{t}$ is the turbulent viscosity, and $u_{i}$ is the average velocity. When *i* = *j*, $\delta_{ij}$ = 1; when i≠j, $\delta_{ij}$ = 0. $C_{\mu }$ is an empirical constant.

(2) $$\left\{\begin{array}{ccc} -\rho \bar{u_{i}^{{}^{\prime }}u_{j}^{{}^{\prime }}} & = & \mu_{t}\left(\frac{\partial u_{i}}{\partial x_{j}}+\frac{\partial u_{j}}{\partial x_{i}}\right)-\frac{2}{3}\left(\rho k+\mu_{t}\frac{\partial u_{i}}{\partial x_{i}}\right)\delta_{ij},\\ \mu_{t} & = & \rho C_{\mu }\frac{k^{2}}{\varepsilon },\\ k & = & \frac{\bar{u_{i}^{{}^{\prime }}u_{j}^{{}^{\prime }}}}{2},\\ \varepsilon & = & \frac{\mu }{\rho }\bar{\left(\frac{\partial u_{i}^{{}^{\prime }}}{\partial x_{k}}\right)\left(\frac{\partial u_{i}^{{}^{\prime }}}{\partial x_{k}}\right)}. \end{array}\right.$$

**Figure 1:**
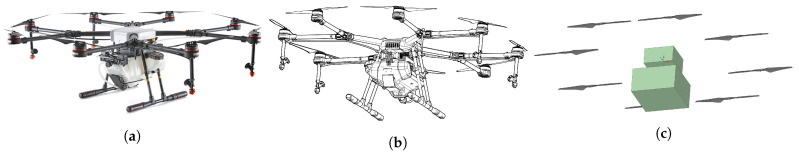
(**a**,**b**) show the UAV physical object and 3D model, respectively. (**c**) shows the simplified UAV model. The simplified UAV model consists of eight 2170 propellers, a control module shell, and a water tank.

The simulation and experiment to obtain the lift curve, wind field distribution, and droplet deposition distribution of UAVs (MG-1S, DJ-Innovation Technology Co., Ltd., Shenzhen, China) are designed and conducted. This process is performed to verify the effectiveness of the CFD simulation adopted in this study. The simulation parameters are set in accordance with the experiment. An accurate 3D model of UAV plays a key role in CFD simulation, but the complex structure leads to difficulties in modeling and boundary conditions. As shown in Figure 1b, simplifying the UAV structure, which has no obvious effect on the wind field, is necessary because the shape of the UAV is a complex curved surface. The simplified result is shown in Figure 1c, and the simplified rules are as follows.

- Remove the landing gears, nozzles, arms, and other small structures.
- Omit the wires, mainboard, fixings, and other internal items.
- Retain the propellers, control module shell, and water tank.
- Simplify the complex surface into a flat surface.

**Figure 2:**
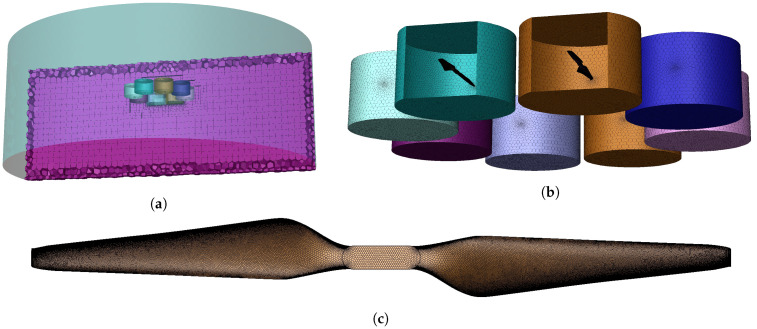
(**a**) shows the whole computing domain, which consists of 48,851,963 nodes. The eight dynamic regions are shown in (**b**). (**c**) shows the propeller, which consists of approximately 4900 surfaces. The interface meshes are refined for accurate calculation.

In accordance with the SST *k*–ω turbulence model, momentum, turbulent kinetic energy, and specific dispatch rate are all second-order upwind. In the 3D coordinates, the positive *X*−direction motion is forward, the positive Y−direction motion is the rise, and the positive Z−direction motion is the right offset. The UAV is located at the *Y* = 2 m plane, and its Y−direction projection point is the center of the plane (*X* = 0, *Y* = 0 m). As shown in Figure 2a, the whole calculation domain is a cylinder with a radius of 4.5 m and a height of 3 m. The size and position of UAV meshes refer to the real UAV settings in Figure 1a. As shown in Table 2, several regions are set up in the model to simulate the hovering state of UAV. The lift calculation and two-phase flow simulation are completed by using the sliding mesh method [59].

- The dynamic region represents the area where the propeller rotates.
- The empty region represents the eight propellers, control module shell, and water tank.
- The static region represents the area affected by the UAV wind field.

### 2.2. Comparison of Lift Curve and Wind Field Distribution

**Figure 3:**
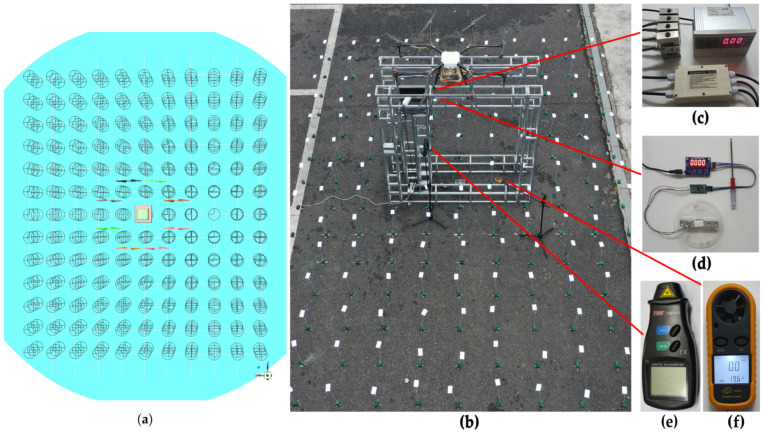
Data acquisition of lift and wind velocity. (**a**) shows the sample points in the simulation. (**b**) shows the scene in the experiment. (**c**–**f**) show the positive and negative force sensors of UAV’s landing gears, laser tachometer, and anemometer.

The sample points are used to obtain the simulation data shown in Figure 3a, and the sample points in the experiment are used to verify the reliability of simulation data shown in Figure 3b. In the simulation, the wind velocity sample area is composed of 429 sample points with an interval of 0.5 m. They are distributed on three planes with *Y* = {0.5, 1.0, 1.5 m}. Each plane consists of 11 × 13 (5 m × 6 m) sample points. The sample points in the experiment are set in accordance with their position in the simulation. Figure 3c shows the force sensor (XSB6-AHK1R4S2V0, Tianhe Automation Instrument Co., Ltd., Shanghai, China) used to measure the positive force of UAV’s landing gear, with a range of 0.0001~1000 N and an error of 0.05%. Figure 3d shows the force sensor (HX711, Runeskee Luojia Technology Co., Ltd., Shenzhen China) for measuring the negative force of UAV landing gear, with a range of 0.01~100 N and an error of 0.01 N. Figure 3e shows the laser tachometer (TA8146A, Terans Electronic Co., Ltd., Suzhou, China) used to measure the real-time speed of eight propellers, with a range of 2.5~9999 rpm and an error of 0.5 rpm. Figure 3f shows the digital anemometer (GM816, Jumaoyuan Technology Co., Ltd., Shenzhen, China), with a range of 0.1~30 m/s and an error of 0.1 m/s. This anemometer is used to measure the wind velocity at the sample points.

**Figure 4:**
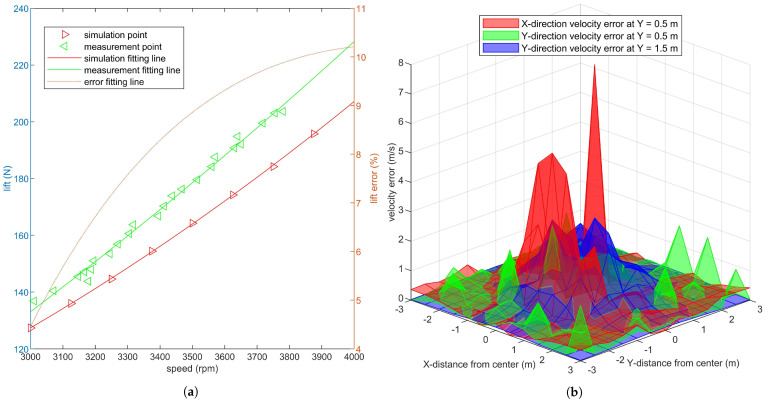
Lift curve and wind field error between the simulation and experiment. (**a**) shows the lift sample points and their fitting curves at different speeds and the error between the experiment and simulation. (**b**) shows the error of X−direction and Y−direction velocities at each sample point on *Y* = {0.5, 1.5 m} planes between the simulation and experiment.

The experiment site is an open space in front of the engineering building of Sichuan Agricultural University. The environment temperature is 23 ${}^{\circ }$C, the relative humidity is 76%, and the environment wind velocity is less than 0.5 m/s. In the propeller-speed-lift verification experiment, the throttle lever in the remote control is continuously raised, the speed of eight propellers rises, and the positive and negative force values on the UAV landing gear and the rotational speeds of eight propellers are recorded. Lift measurement points shown in Figure 4a are obtained by correlating the displayed values of force sensors and the average values of eight propeller speeds. The simulation points shown in Figure 4b are calculated by Fluent.

(3) $$error=\frac{|F_{simul}-F_{expri}|}{F_{expri}}\times 100\%.$$

As shown in Figure 4a, an approximately linear monotone increasing relation is found between lift and speed in the normal working range of 3000~4000 rpm. The experimental results show that the quadratic curve can obtain a small fitting error with a low calculation amount, which is consistent with the conclusion of Li et al. [60,61]. The error fitting curve is calculated in accordance with Formula (3) from the values of the two curves at the same speed. At the same propeller speed, the simulation lift is smaller than the experimental lift. With the increase in speed, the error increases, but the change trend decreases. The extreme values of error appear at the two endpoints of 3000, 4000 rpm, which are 4.41% and 10.23%, respectively.

**Figure 5:**
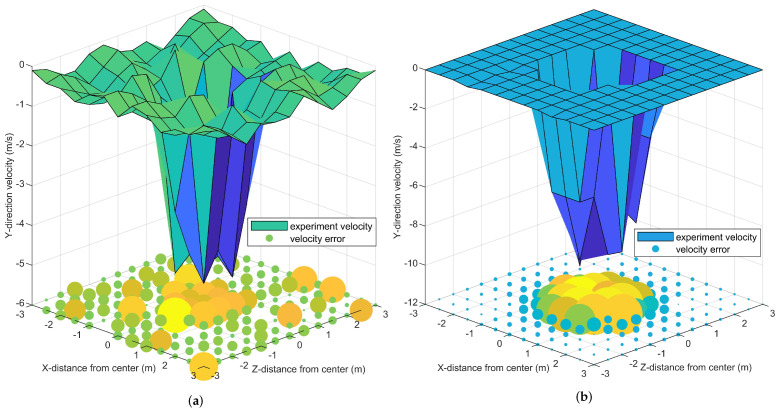
Wind field distribution in the simulation and experiment. (**a**,**b**) show the distributions of wind velocity in Y−direction at the planes of *Y* = {0.5, 1.5 m} and the absolute value of wind velocity error between the simulation and experiment.

The X−direction and Y−direction wind velocities of 429 sample points under 3500 rpm propeller are recorded to verify the realness of simulation wind field. The Y−direction wind velocity and its error figure of *Y* = {0.5, 1.5 m} sample planes are drawn, as shown in Figure 5a,b, respectively. Figure 4b shows the difference in wind velocity between the simulation and experiment, and different colors are used to indicate different heights and directions. The error is represented by the absolute value of wind velocity difference between the simulation and experiment.

From the comparison of Figure 4b and Figure 5a,b, the following conclusions can be obtained.

- The negative Y−direction velocity is dominant in the downwash wind field. The airflow produces ground effect after contacting the ground and spirals out with the UAV projection on the ground as the center, similar to a reverse tornado.
- The maximum Y−direction velocities of *Y* = {0.5, 1.5 m} planes are 5.3 and 10.3 m/s, respectively. The main source of wind power is the propeller rotation. With the airflow away from the propeller, the wind velocity gradually decreases.
- The airflow Y−direction velocity at *Y* = 1.5 m is concentrated in the area of −1.5 m < *X*, *Z* < 1.5 m, which is directly under the propeller. However, the Y−direction velocity distribution in the plane with *Y* = 0.5 m is uniform, and the airflow at the plane margin is slightly disturbed. This condition shows that the downwash wind field tends to expand.
- The airflow Y−direction velocity has an obvious error of 1~2 m/s under the propeller and near the ground, and the maximum value error is 2.88 m/s. The error in other areas is within the range of 1 m/s. The error of X−direction wind velocity in *Y* = 0.5 m plane is large due to the existence of ground effect. This phenomenon indicates that the downwash wind field is complex and changeable when it contacts the ground.

These conclusions are consistent with Yang et al.’s experiment [31,32], showing that the downwash wind field error generated by CFD is acceptable under the case of farmland plant protection.

### 2.3. Comparison of Droplet Deposition Distribution

**Figure 6:**
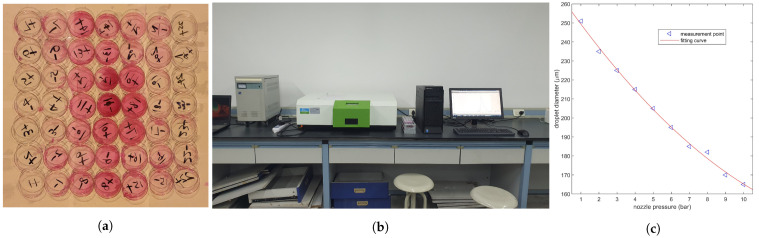
(**a**) shows the solutions collected on the sheets. (**b**) presents the fluorescence spectrophotometer. (**c**) indicates the characteristic curve of nozzle ($D=0.375\times P^{2}-13.48\times P+262.5$, and root mean square error (RMSE) = 1.824).

According to Sheng et al.’s method [49], 0.1 g/L Rhodamine-B solution (analytically pure) was used to spray rather than pesticide. As shown in Figure 3b, polyethylene terephthalate (PET) sheets (85.60 mm × 53.98 mm) were placed at 154 (11 × 14) sample points 0.3 m above the ground, and the spacing between them was 0.5 m. The UAV is equipped with four nozzles (XR11001VS, Tejet Spray Technology Co. Ltd., Tianjin, China), which are located on two sides of the UAV (*X* = ±265, *Z* = ±711 mm). The relationship between nozzle pressure and droplet diameter curve in accordance with the user manual is shown in Figure 6c. Figure 6b shows the fluorescence spectrophotometer (LS55, PerkinElmer Inc., Waltham, MA, USA) used for measuring the solution concentration. The experiment process is as follows.

**Figure 7:**
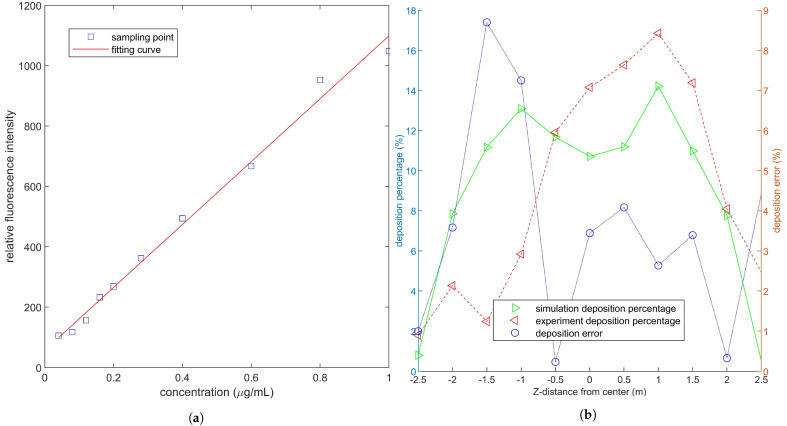
(**a**) shows the standard curve of the relationship between relative fluorescence intensity and solution concentration. (**b**) shows the droplet deposition ratio in the experiment and simulation, and the error between them.

- Standard Rhodamine-B solutions (0.04, 0.08, 0.12, 0.16, 0.2, 0.28, 0.4, 0.6, 0.8, 1 μg/mL) were prepared. The solutions were irradiated at the excitation wavelength of 554 nm. The concentration and intensity were recorded and fitted to the standard curve shown in Figure 7a by using a linear function. The standard curve was RFI=1.0423×C+0.0567, and the coefficient of determination ($R^{2}$) was 0.9960.
- Under the propeller speed of 3500 rpm, four nozzles continuously worked for 5 s to spray 0.1 g/L Rhodamine-B solution. The white PET sheet was washed with secondary distilled water within 20 min until no obvious red dye residue was found after the droplet deposition. Part of the collected solutions after washing is shown in Figure 6a.
- The collected solutions were diluted to 20 mL, and the foil was used to avoid light degradation during transportation. The relative fluorescence intensity of each constant temperature solution in the spectrophotometer is recorded, and the droplet deposition is calculated in accordance with Figure 7a and Formula (4).

(4) $$\beta_{dep}=\frac{\rho_{sampl}V_{sampl}}{\rho_{spray}A_{col}}.$$

In Formula (4), $\beta_{dep}$ is the amount of droplet deposition in mL/cm${}^{2}$. $\rho_{sampl}$ is the concentration of the solution washed from the PET plate in μg/mL and can be obtained from the standard curve. $V_{sampl}$ is the volume of collected solution in mL. $\rho_{spray}$ is the concentration of Rhodamine-B in the spray solution in μg/mL. $A_{col}$ is the area of PET sheet used to collect droplets in cm${}^{2}$.

The absolute value of the difference between the experiment and simulation deposition percentages is plotted in Figure 7. The droplet deposition curve is axisymmetric because the environment wind velocity is set to zero in the simulation, and the maximum deposition percentage is 13.1% at *Z* = 1 m. The outdoor experiment’s deposition curve slightly deviates to the positive Z−direction due to the effect of crosswind and non vertical nozzles [19]. The maximum simulation error occurrs at *Z* = −1.5 m, which is 8.7%, and the error of other areas is within 5%. This condition indicates that the strong downwash wind field reduces the effect of crosswind on the deposition, which is consistent with the experimental results of Yang et al. [44]. The small error indicates that the CFD simulation can be applied to predict the droplet deposition distribution of plant protection UAV.

## 3. Simulation Deposition Data Acquisition

### 3.1. Effect Factors of Deposition Distribution

As shown in Table 3, multiple factors affect the spray droplet deposition of UAV. These factors can be divided into three categories: flight state (propeller speed, pitch angle, flight height), environment state (wind velocity, environment temperature, environment humidity), and nozzle state (nozzle flow, droplet diameter, propeller nozzle distance). The deposition characteristic caused by different plant protection methods is different. Thus, the parameter range in the simulation is set in accordance with the general working state of MG-1S UAV. The nozzle type is fan-plane pressure nozzle, and the injection material is liquid water. The diameter distribution of the droplets emitted by the injection is Rosin-Rammler, the mean value is our preset VMD, and the maximum and minimum diameters are 260 and 140 μm, respectively. The DPM boundary on the top and side surfaces of the cylindrical wind field is particle escape, the bottom surface is particle trap, and the DPM boundary of UAV fuselage is particle reflection. Coupled heat-mass solution, breakup, coalescence, and stochastic collistion of the physical model are activated. The deposition ratio is obtained by dividing the number of particles collected per area by the number of particles ejected from the nozzle. This process is performed to facilitate the calculation of spray droplet deposition volume in the simulation. The effect of nine factors on the droplet deposition ratio at different states is recorded through orthogonal simulation.

The droplets are affected by initial state, gravity, wind, and evaporation after they are ejected from the nozzle. Gravity is the common mode of droplet motion and the driving factor of negative Y−direction motion. Most of the droplets deposit on the leaves or on the ground due to the existence of gravity. The initial state has a considerable effect on the deposition position, especially when the environment wind velocity is small. The nozzle gives the velocity component of the droplet horizontal movement and can change the spray coverage range and droplet deposition per area. Wind causes the transfer of deposition center, leading to nonuniform deposition. Evaporation is the main cause of pesticide loss, thereby directly affecting the effect of plant protection with small-size droplets [20].

### 3.2. Comparison of Effect Factors

**Figure 8:**
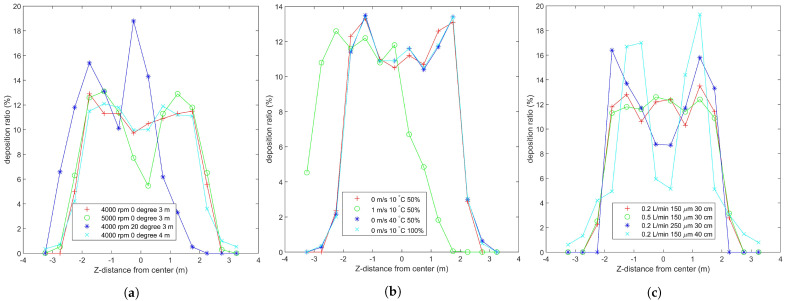
Deposition ratio curve of distance from the center in different states. (**a**–**c**) shows the droplet deposition ratio under different flight, environment, and nozzle states, which correspond to Figure 9, Figure 10 and Figure 11, respectively.

**Figure 9:**
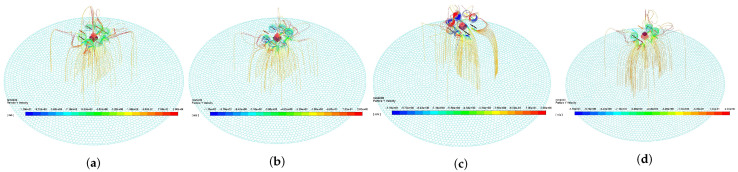
Droplet deposition trajectory under different flight states. The track color indicates the droplet velocity in Y−direction. (**a**) shows the UAV flying at 4e3 rpm, without tilt, and 3 m above the ground. (**b**) shows the UAV flying at 5e3 rpm without tilt, and 3 m above the ground. (**c**) shows the UAV flying at 4e3 rpm, 20${}^{\circ }$ tilt, and 3 m above the ground. (**d**) shows the UAV flying at 4e3 rpm, without tilt, and 4 m above the ground.

The four curves in Figure 8a correspond to the four figures in Figure 9, which reflect the effect of droplet movement and deposition characteristic under different flight states. From the comparison between Figure 9a,b, the faster the propeller speed is, the stronger the downwash wind field, and many droplets are drawn into the propeller by the airflow. The large negative Y−direction velocity leads to the decrease in the spray divergence [24], and its center is the Y−direction projection of the nozzle. Figure 9c shows the droplet trajectory of the tilted UAV. The tilted wind field drives the droplets to move in the negative Z−direction, making the deposition ratio nonuniform. As shown in Figure 9d, the deposition distribution at the margin slightly increases with the increase in height. This condition may be due to the trajectory change caused by the breakup of droplets [19].

**Figure 10:**
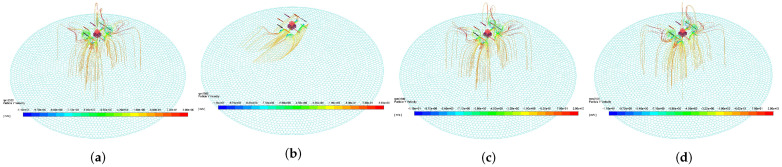
Droplet deposition trajectory under different environment states. The track color indicates the droplet velocity in Y−direction. (**a**) shows the environment state of 0 m/s wind velocity, 10 ${}^{\circ }$C temperature, and 50% relative humidity. (**b**) shows the environment state of 1 m/s wind velocity, 10 ${}^{\circ }$C temperature, and 50% relative humidity. (**c**) shows the environment state of 0 m/s wind velocity, 40 ${}^{\circ }$C temperature, and 50% relative humidity. (**d**) shows the environment state of 0 m/s wind velocity, 10 ${}^{\circ }$C temperature, and 100% relative humidity.

The four curves in Figure 8b correspond to the four figures in Figure 10, which reflect the effect of droplet movement and deposition characteristic under different environment states. On the one hand, Figure 10b shows that the droplets have obvious offset under the effect of negative X−direction wind [20]. When the droplet moves out of the downwash wind field, the offset phenomenon is obvious. This phenomenon shows that the downwash wind field is conducive to the uniform deposition of droplets. On the other hand, environment temperature and humidity have minimal effect on the distribution characteristics, as shown in Figure 8b. Their curves mostly coincide, but they are mainly reflected in the evaporation loss of droplets [26].

**Figure 11:**
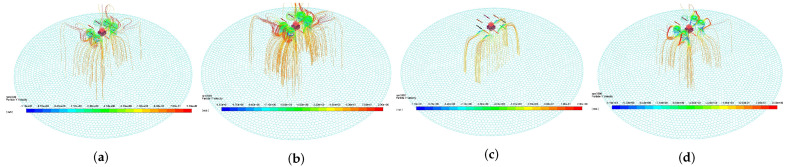
Droplet deposition trajectory under different nozzle states. The track color indicates the droplet velocity in Y−direction. (**a**) shows the nozzle working at a flow rate of 0.2 L/min, droplet diameter of 150 μm, and a distance of 30 cm from the propeller. (**b**) shows the nozzle working at a flow rate of 0.5 L/min, droplet diameter of 150 μm, and a distance of 30 cm from the propeller. (**c**) shows the nozzle working at a flow rate of 0.2 L/min, droplet diameter of 250 μm, and a distance of 30 cm from the propeller. (**d**) shows the nozzle working at a flow rate of 0.2 L/min, droplet diameter of 150 μm, and a distance of 40 cm from the propeller.

The four curves in Figure 8c correspond to the four figures in Figure 11, which reflect the effect of droplet movement and deposition characteristic under different nozzle states. As shown in Figure 11b, the nozzle flow is mainly reflected in the deposition volume rather than the ratio. However, the small droplet diameter is easy to be affected by wind, and the large droplet leads to nonuniform deposition, as shown in Figure 11c. Therefore, finding a suitable droplet diameter is necessary [18]. The distance between the propeller and the nozzle is related to the distribution of downwash wind field. A distance corresponding to the downwash wind field promotes the formation of a uniform deposition distribution [27].

The propeller speed, pitch angle, wind velocity, and droplet diameter play a major role in the distribution characteristics of droplet deposition. In the case of wind, the flight height should be reduced and the droplet diameter should be increased [28]. However, under strong wind, the UAV plant protection operation should be avoided to ensure safety and spray effect. At extreme temperatures, such as greater than 40 ${}^{\circ }$C or less than 0 ${}^{\circ }$C, spray is easy to evaporate or freeze. If necessary, dissolve additives or increase the flow rate of nozzle, but the best way is to avoid flying [62].

From the deposition ratio curve in Figure 8, the different spray states are characterized by high and flat center and less steep sides. This condition provides theoretical support for the uniformity of the variable spray system. The simulation and experiment are repeatedly compared, and the related work of predecessors is consulted. The simulation data can be used to represent the effect of various states on droplet deposition distribution characteristics under the normal work condition of MG-1S UAV.

In accordance with the range in Table 3, the data set is generated through calculation or interpolation in the simulation. Each record includes 12 parameters (nine variables that affect the spray droplet deposition distribution characteristics, X−direction and Z−direction coordinates of the sample points distributed in the range of 3 m × 3 m, and droplet deposition ratio at the sample points). The data set is divided into train and test sets, and the quantity ratio is 8:2. The data set is read and trained through regression analysis after the following data cleaning. The output value of regression model is used in the path planning of plant protection UAV.

- The droplet collection surface is the bottom of the static region, and the droplet that does not reach the surface within the convergence time is ignored.
- The droplet deposition rate in the data set is accurate to three decimal places, and the redundant number is ignored to unify the specification for data transmission.
- The droplet deposition rate of the same spray state is only recorded once to avoid the regression analysis bias caused by the data size [63].
- After removing some misleading deposition rate records (less than zero or greater than one), the values are normalized using Formula (5).

(5) $$X=\left(0.98-0.02\right)\left(\frac{X_{i}-X_{min}}{X_{max}-X_{min}}\right)+0.02.$$

## 4. Regression Analysis

### 4.1. Introduction of Regression Algorithms

CFD simulation can predict the distribution characteristics of spray droplet deposition within the acceptable error range. However, some problems, such as complex model, large amount of calculation, and long time consumption, are found in the simulation. These problems make it difficult to realize the real-time prediction with variable spray system. Multiple regression analysis is a statistical analysis method used to determine the quantitative relationship between two or more variables. Regression analysis can be described as follows. For a given training sample $D=\left\{\left(x_{1},y_{1}\right),\left(x_{2},y_{2}\right),...,\left(x_{m},y_{m}\right)\right\},y_{i}\in R$, we aim to learn a fitting model similar to Formula (6), so that f(x) and *y* are as close as possible, and w and *b* are the model parameters to be determined. Support vector regression (SVR) and back propagation neural network (BPNN) are widely used regression models and are proven to be reliable and efficient in many fields [42,43,44].

(6) $$\begin{array}{ccc} f(x) & = & w^{T}x+b. \end{array}$$

SVM is proposed for binary classification problem, and SVR is an important branch of SVM in regression analysis [64]. SVR assumes that a maximum deviation of ϵ between f(x) and *y* can be tolerated, which is equivalent to constructing a 2ϵ-wide isolation belt centered on f(x). If the training samples fall into this interval, the prediction results are considered to be correct. SVR can be expressed as Formula (7).

(7) $$\begin{array}{ccc} \underset{w,b}{min}\frac{1}{2}{∥w∥}^{2} & + & C\sum_{i=1}^{m}l_{\epsilon }\left(f\left(x_{i}\right)-y_{i}\right), \end{array}$$

where *C* is the regularization constant, and $l_{\epsilon }$ is the ϵ−insensitive loss function, which can be expressed as Formula (8).

(8) $$\begin{array}{cc} l_{\epsilon }\left(z\right)= & \left\{\begin{array}{cc} 0, & if\;|z|⩽\epsilon ,\\ |z|-\epsilon , & otherwise. \end{array}\right. \end{array}$$

The SVR dual problem shown in Formula (9) can be obtained by introducing Lagrange multiplier $\alpha_{i}⩾0$, ${\hat{\alpha }}_{i}⩾0$ and relaxation variable $\xi_{i}⩾0$, ${\hat{\xi }}_{i}⩾0$. The Karush–Kuhn–Tucker (KKT) condition of Formula (10) should be satisfied in the solving process.

(9) $$\begin{array}{cc} \underset{\alpha ,\hat{\alpha }}{max} & \sum_{i=1}^{m}y_{i}\left({\hat{\alpha }}_{i}-\alpha_{i}\right)-\epsilon \left({\hat{\alpha }}_{i}+\alpha_{i}\right)\\ \; & -\frac{1}{2}\sum_{i=1}^{m}\sum_{j=1}^{m}\left({\hat{\alpha }}_{i}-\alpha_{i}\right)\left({\hat{\alpha }}_{j}-\alpha_{j}\right)x_{i}^{T}x_{j}.\\ s.t. & \sum_{i=1}^{m}\left({\hat{\alpha }}_{i}-\alpha_{i}\right)=0,\\ \; & 0⩽\alpha_{i},{\hat{\alpha }}_{i}⩽C. \end{array}$$

(10) $$\begin{array}{cc} KKT. & \left\{\begin{array}{c} \alpha_{i}\left(f\left(x_{i}\right)-y_{i}-\epsilon -\xi_{i}\right)=0,\\ {\hat{\alpha }}_{i}\left(y_{i}-f\left(x_{i}\right)-\epsilon -\xi_{i}\right)=0,\\ \alpha_{i}{\hat{\alpha }}_{i}=0,\xi_{i}{\hat{\xi }}_{i}=0,\\ \left(C-\alpha_{i}\right)\xi_{i}=0,\left(C-{\hat{\alpha }}_{i}\right){\hat{\xi }}_{i}=0. \end{array}\right. \end{array}$$

Sequential minimal optimization can be used to decompose the original quadratic programming problem into subproblems with only two variables and solve the subproblem until all variables meet the KKT condition [65]. SVR can be expressed as Formula (11), where $K(x,x_{i})$ is the kernel function.

(11) $$\begin{array}{c} f\left(x\right)=\sum_{i=1}^{m}\left({\hat{\alpha }}_{i}-\alpha_{i}\right)K\left(x,x_{i}\right)+b. \end{array}$$

Formula (12) shows the kernel functions used in regression analysis. They are linear kernel, polynomial kernel, radial basis kernel, and sigmoid kernel. Linear kernel has strong interpretability and fast solution, but it can only be used to solve linear separable problems. The polynomial kernel makes the original data linearly separable by lifting the dimension and integrates the experience by setting the polynomial degree subjectively. However, this kernel is extremely complex for large order of polynomial parameters. Radial basis function has only one parameter and can be mapped to infinite dimensional space. However, it has poor interpretability, slow calculation speed, and easy over fitting. The SVR model with sigmoid function as kernel function is a multilayer perceptron neural network model. Similar to other neural networks, the number of hidden layers and the number of nodes in each hidden layer need to be determined by expert experience in advance. The weight value of each network layer is determined by reducing the expected error during training.

(12) $$\begin{array}{cccc} K_{Linear}\left(x_{i},x_{j}\right) & = & x_{i}^{T}x_{j}+C, & \\ K_{Polynomial}\left(x_{i},x_{j}\right) & = & {\left(x_{i}^{T}x_{j}\right)}^{d}, & d⩾1,\\ K_{RBF}\left(x_{i},x_{j}\right) & = & exp(-\frac{1}{2\sigma^{2}}∥x_{i}-x_{j}{∥}^{2}), & \sigma >0,\\ K_{sigmoid}\left(x_{i},x_{j}\right) & = & tanh(\beta x_{i}^{T}x_{j}+\theta ), & \beta >0,\theta <0. \end{array}$$

Back propagation is a common method used to train ANNs in combination with optimization methods, such as gradient descent [66]. The gradient of loss function is calculated for all weights in the network, and the gradient is fed back to the optimization method to update the weights for minimizing the loss function. Back propagation requires that the desired output of each input value must be known and is used to calculate the loss function gradient. Therefore, back propagation is usually considered as a supervised learning method and requires that the activation function of nodes is differentiable. Wang et al. proved that BPNN can fit a continuous function of arbitrary complexity only by a hidden layer containing sufficient neurons [67].

(13) $$x_{i}=\frac{x_{i}-min\left(x\right)}{max(x)-min(x)}.$$

(14) $$\begin{array}{ccc} \alpha_{h} & = & \sum_{i=1}^{d}v_{ih}x_{i},\\ \beta_{j} & = & \sum_{h=1}^{q}w_{hj}b_{h}. \end{array}$$

(15) $${\hat{y}}_{j}^{k}=f\left(\beta_{j}-\theta_{j}\right).$$

(16) $$g_{j}={\hat{y}}_{j}^{k}\left(1-{\hat{y}}_{j}^{k}\right)\left(y_{j}^{k}-{\hat{y}}_{j}^{k}\right).$$

(17) $$e_{h}=b_{h}\left(1-b_{h}\right)\sum_{j=1}lw_{hj}g_{j}.$$

(18) $$\begin{array}{ccc} \Delta w_{h}j & = & \eta g_{j}b_{h},\\ \Delta \theta_{j} & = & -\eta g_{j},\\ \Delta v_{i}h & = & \eta e_{h}x_{i},\\ \Delta \gamma_{h} & = & -\eta e_{h}. \end{array}$$

(19) $$E=\frac{1}{2m}\sum_{k=1}^{m}\sum_{j=1}^{l}{\left({\hat{y}}_{j}^{k}-y_{j}^{k}\right)}^{2}.$$

Algorithm 1 shows the BPNN algorithm workflow. The network contains *d* input neurons, *q* hidden neurons, and *l* output neurons. The threshold of the *j* th neuron in the output layer is $\theta_{j}$, and that of the *h* th neuron in the hidden layer is $\gamma_{h}$. The connection weight between the *i* th neuron in the input layer and the *h* th neuron in the hidden layer is $v_{ih}$, and that between the *h* th neuron in the hidden layer and the *j* th neuron in the output layer is $w_{hj}$. The output of the *h* th hidden layer neurons is $b_{h}$. The sigmoid function shown in Formula (12) is assumed to be used by all neurons.
**Algorithm 1** BPNN algorithm workflow**Input:**      Training set: $D=\{\left(x_{1},y_{1}\right),\left(x_{2},y_{2}\right),...,\left(x_{m},y_{m}\right)\}$, $y_{i}\in R$;    Learning rate: η;    Initial neuron parameters: w, v, θ, γ.**Output:** Effective neuron parameters: w, v, θ, γ.1:The samples and network parameters are initialized according to Formula (13);2:**for** all $(k_{k},y_{k})\in D$ **do**3:      The input β of the output neuron is calculated according to Formula (14);4:      The output ${\hat{y}}_{j}^{k}$ of the current sample is calculated according to Formula (15);5:      The gradient $g_{j}$ of the neuron in the output layer is calculated according to Formula (16);6:      The gradient $e_{h}$ of neurons in the hidden layer is calculated according to Formula (17);7:      The Neuron parameters are updated according to Formula (18);8:      **if** The cumulative error calculated by Formula (19) is in accordance with the expectation **then**9:          Break;     10:**return** The neuron parameters.

### 4.2. Comparison of Regression Models

As shown in Formula (20), RMSE, mean absolute error (MAE), and $R^{2}$ were used to measure the performance of regression analysis results. In Formula (20), $d_{i}$ is the actual value, $\bar{d}$ is the arithmetic mean of the actual value *d*, *d* is the vector representation of $d_{i}$, $y_{i}$ is the predicted value, and *n* is the sample number. The range of RMSE is [0, +*∞*), and the RMSE is equal to 0 when the predicted value is completely consistent with the real value, that is, the perfect model. The greater the error is, the greater the value. An MAE of 0% is a perfect model, and an MAE greater than 100% is a poor model. The value range of $R^{2}$ is [0, +1]. The closer $R^{2}$ is to 1, the better the explanation of independent variable to dependent variable in regression analysis.

(20) $$\begin{array}{ccc} RMSE & = & \sqrt{\frac{1}{n}\sum_{i=1}^{n}{\left(d_{i}-y_{i}\right)}^{2}},\\ MAE & = & \frac{1}{n}\sum_{i=1}^{n}\left|d_{i}-y_{i}\right|,\\ R^{2} & = & 1-\frac{\sum_{n}^{i=1}{\left(d_{i}-y_{i}\right)}^{2}}{\sum_{n}^{i=1}{\left(d_{i}-\bar{d}\right)}^{2}}. \end{array}$$

Table 4 shows the performance of multiple regression models on the simulation data set. For SVR, the parameters are kernel type, and the parameters in brackets are the degrees of polynomial kernel. For BPNN, the parameter is the number of hidden layers, and the number of neurons in each layer is in brackets. The number of neurons in each layer is selected from 1 to 3001 by the program automatically to minimize the criterion of RMSE-test. The training process and results show that SVR can quickly fit the data, and few parameters are set manually. However, the optimal SVR (sigmoid) has an error of approximately 10% on the simulation data set. The BPNN can decompose the data characteristics and fit the complex data with multiple layers and units. The minimum error is approximately 6.5%, although the iterative process and parameter selection cost considerable time and computing resources. This result is consistent with the conclusion of Niazian et al. [50]. The variable spray system selects BPNN with five hidden layers (991, 833, 794, 767, 761).

## 5. Variable Spray System

### 5.1. Design of Spray System

**Figure 12:**
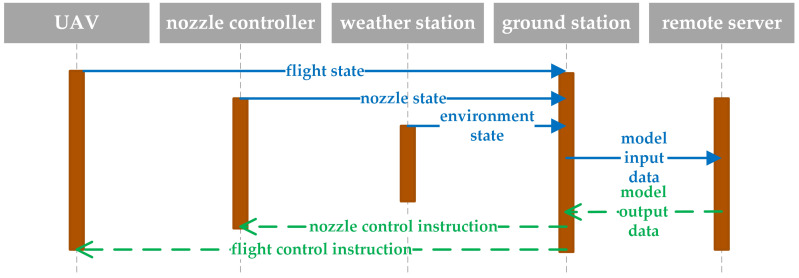
Sequence diagram of variable spray system.

Figure 12 shows the working sequence diagram of the variable spray system in this study. The state perception and control function of the UAV and nozzle are provided by MG-1S, and the environment state perception is provided by the weather station (6152EU, Davis Instruments Co. Ltd, Hayward, CA, USA). The ground station is composed of remote controller (A14-057N1A, DJ-Innovation Technology Co., Ltd., Shenzhen, China), weather station console (Vantage Pro2, Davis Instruments Co. Ltd, Hayward, CA, USA), and portable computer (ThinkPad P53, Lenovo Co. Ltd, Beijing, China). The regression analysis model is deployed on the remote server. In the design of communication network, the Internet of Things protocol, such as long range network, has the characteristics of long distance and low power consumption, which is widely used in PA. However, the low bandwidth limits the transmission of large amounts of data in the experiment [68]. Therefore, the communication mode between the UAV and ground station is 2.4 GHz wireless local area network, and the communication mode between the ground station and remote server is 4G wireless data terminal.

**Figure 13:**
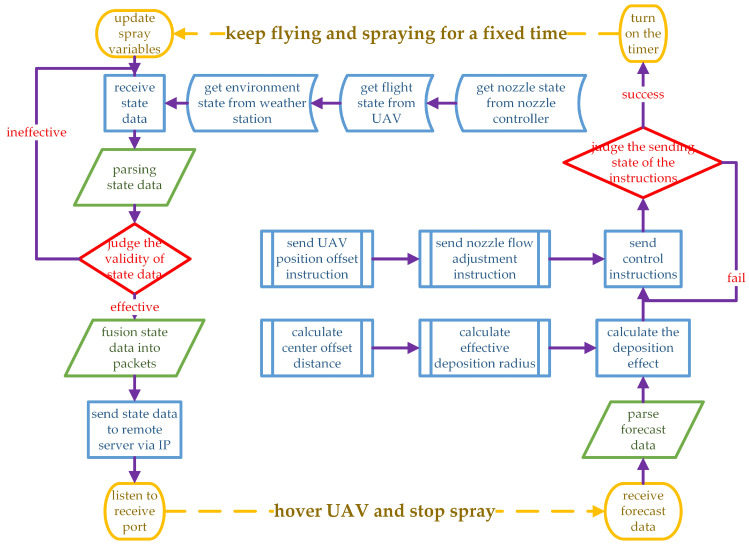
Data receiving, processing, and transmission flow chart of ground station.

Figure 13 shows the workflow of ground station. The ground station is the intermediary between the perception and execution modules and is responsible for data transfer, parameter calculation, and instruction sending. The parameters calculated by the ground station are center offset distance and effective deposition radius, which are defined as follows.

- Center offset distance: The X−direction and Z−direction components of the distance between the approximate pattern center of droplet deposition distribution and the vertical projection of UAV in {m, m}.
- Effective deposition radius: The droplet deposition ratio curve is drawn with the deposition ratio as the ordinate and the sample points on two sides of the UAV flight route as the abscissa. Two points are found on two sides of the curve, and the deposition ratio of these points is half of the maximum [69]. Half the distance between two points is the effective deposition radius in m.

When the UAV approaches the boundary of the operation area, the ground station starts to work. The ground station receives the state data from three sensor modules and judges the validity. Invalid conditions include low residual power, less residual pesticide, weak signal, bad working environment (wind speed, temperature), and pump failure. If the operation conditions are met, the state data are sent to the remote server through IP address after normalization and packaging. During waiting for remote server response, the UAV should be hovering and stop spraying.

After receiving the prediction data from the remote server, the effective deposition radius $\left(R_{e}\right)$ and center offset distance $\left(D_{\left(X,Y\right)}\right)$ are calculated using Formula (21). The sample area consists of *m* sample points, which is 7 × 7 in the experiment. $P_{X}$ is the X−direction component of sample point location in m, and $P_{Y}$ is the Y−direction component of sample point location in m. $R_{p}$ is the droplet deposition ratio at the sample point in % returned by the remote server. $V_{R}$ is the preset prescription volume of pesticide required by the operation area. $V_{f}$ is the flight velocity, which is obtained by the GPS equipment carried by the UAV. The program uses an empirical constant *c* to fit the effective deposition radius due to the complexity of the curve calculation. UAV position offset $\left(D_{\left(X,Y\right)}\right)$ in m and nozzle flow $\left(F_{N}\right)$ in L/min are sent to the UAV manually. When no reply signal is received from the two executive modules, the ground station shall retransmit the parameters. The UAV position offset should make the current effective deposition radius tangent to the non operation boundary. The nozzle flow should be such that the droplet deposition volume deposited in the effective deposition radius is consistent with the preset prescription volume.

(21) $$\begin{array}{ccc} D_{\left(X,Y\right)} & = & (\sum_{i=1}^{m}\left(P_{Xi}\times R_{pi}\right),\sum_{i=1}^{m}\left(P_{Yi}\times R_{pi}\right)),\\ R_{e} & = & \frac{1}{c}\sum_{i=1}^{m}\left|P_{Yi}\times R_{pi}\right|,\\ F_{N} & = & \frac{V_{R}}{10000}\times 2R_{e}\times \left(V_{f}\times 60\right). \end{array}$$

The UAV and nozzle controller fly at a constant velocity after they receive the parameters and spray until reaching the preset time. The flight, environment, and nozzle states remain unchanged in the fixed time period. After that period, the spray state should be updated, that is, start the new cycle until the boundary of the operation area is reached.

### 5.2. Collection of Experimental Data

**Figure 14:**
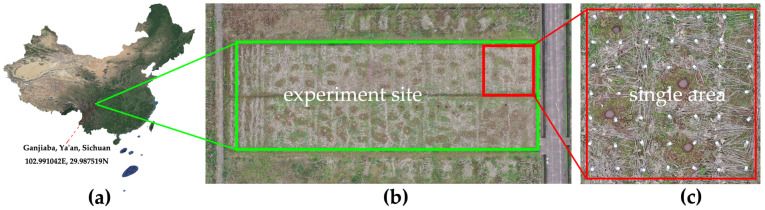
Overview of the experiment site.

The farmland experiment was designed and implemented to verify the effectiveness of the variable spray sysyem. As shown in Figure 14a, the experiment site is the rice and corn planting area in Ganjiaba Base of Sichuan Agricultural University. Ya’an Yucheng District $(102^{\circ }51{}^{\prime }$~$103^{\circ }12{}^{\prime }$ E, $29^{\circ }40{}^{\prime }$~$30^{\circ }14{}^{\prime }$ N) is located in the western edge of Sichuan Basin, with an average altitude of 566 m. The climate type is subtropical monsoon humid climate, with an average annual temperature of 16.1 ${}^{\circ }$C, annual average wind velocity of 1.7 m/s, and annual average humidity of 79%. The experiment time is from 21 to 25 October 2020. The weather is cloudy to overcast, the temperature is 15~25 ${}^{\circ }$C, the southeast wind grade is 0~1 (Beaufort scale), and the relative humidity is 80~100%.

**Figure 15:**
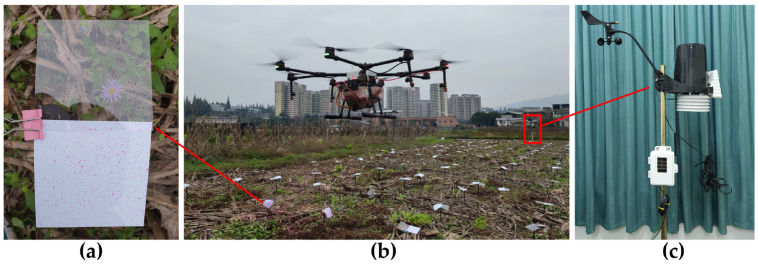
UAV plant protection operation controlled by variable spray system.

The experiment site (16 m × 50 m) shown in Figure 14b is divided into 2 × 6 areas, and 7 × 7 sample points are uniformly set in each area (7 m × 7 m) at 1 m interval, with a total of 12 × 49 sample points. The height of the sample points is 0.3 m, which is the average height of rice leaves. White and transparent PET sheets are fixed above sample points with clamps shown in Figure 15a for the measurement of droplet deposition uniformity and droplet deposition volume. The thickness of PET sheet is 0.3 mm, and its surface is smooth. It is used to simulate the movement of rice leaves. Therefore, the experiment environment is consistent with the natural state of farmland.

**Figure 16:**
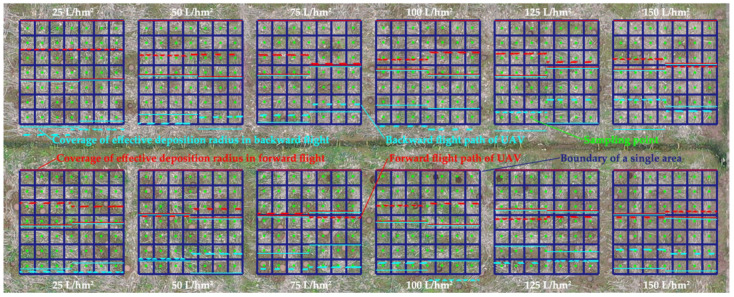
Diagrammatic sketch of flight path and effective deposition radius coverage.

Figure 15b shows the operation of UAV under variable spray system control. Figure 16 shows the UAV flight path restored from the system log and the effective deposition radius coverage predicted by the variable spray system. The red line indicates that the UAV’s flight direction is from right to left, and the blue line indicates that the flight direction is from left to right. The solid line represents the predicted deposition coverage, and the dotted line represents the flight path. The green dot is the location of the sample device shown in Figure 15a. The deposition distribution characteristic of the sample point represents the overall characteristic of the zone surrounded by dark blue lines.

The UAV takes off from the upper-right corner of Figure 16 and moves to the left, which is a forward flight. After reaching the left boundary of the site, the UAV flies from left to right again, which is a backward flight. The flight height is set to 2 m, and the flight velocity is set to 0.5 m/s. The droplet diameter depends on the nozzle pressure in Figure 6c, and the distance between propeller nozzles is 0.3 m. The preset prescription volume of pesticide is the white words in Figure 16. The parameters of propeller speed, pitch angle, and environment state are collected by sensors and sent to the ground station. The wind velocity vector should be added to the flight velocity. The ground station calculates the center offset distance and nozzle flow in accordance with the workflow in Figure 13 and is executed by the UAV and nozzle controller.

The UAV flies four times, including two forward and two backward flights, with a one-way flight distance of 48 m. The variable spray system sends 48 instructions to the UAV for completing the flow adjustment and position offset. Instruction records show that the average time consumption of regression model prediction and telecommunication network transmission is 1.2 and 0.3 s, respectively. The flight state, environment state, nozzle state, center offset distance, and effective deposition radius of 12 instructions are shown in Table 5. After the UAV landing, the PET sheets at the sample points are processed in accordance with Section 2.3, and the droplet deposition volume is obtained. These experimental data are compared with the data recorded in the ground station to verify the effectiveness of the simulation data and the variable spray system.

### 5.3. Discussion of Experimental Results

**Figure 17:**
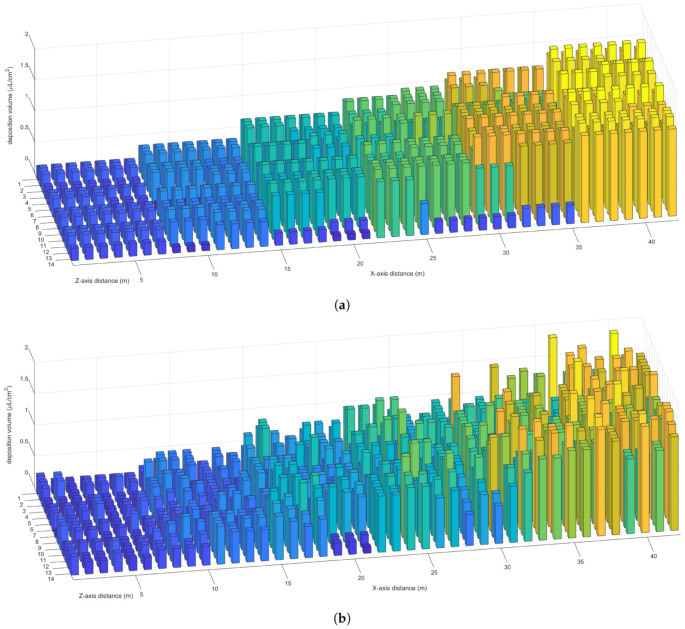

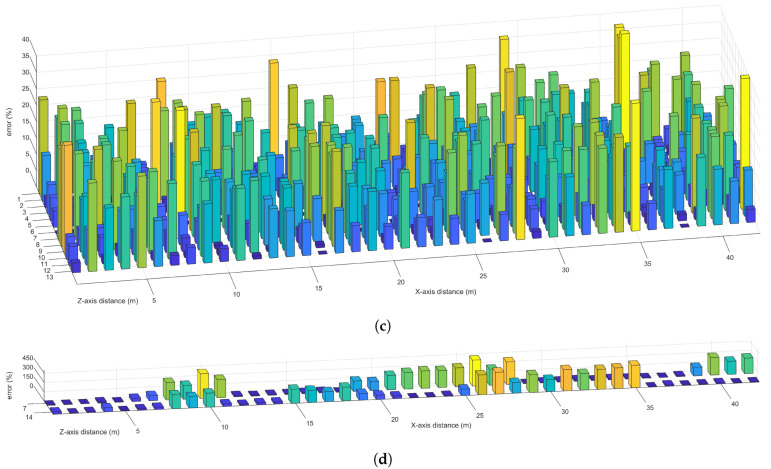
(**a**,**b**) show the predicted and experimental deposition volume per area, respectively. (**c**,**d**) show the prediction error calculated in accordance with Formula (22).

Figure 17a shows the predicted deposition volume at the sample points restored from the state data recorded by the ground station. Figure 17b shows the experimental deposition volume obtained by the spectrophotometer. Figure 17c shows the error in the central part of the area, and Figure 17d shows the error in the margin part of the area due to the large difference in error values. As shown in Table 5, all sensors work normally and send the state data to the ground station continuously. The ground station correctly receives the state data and sents them to the remote server after processing. The nozzle flow and UAV position offset are calculated by the ground station in accordance with the data returned by the remote server, as shown in Figure 17a. As shown in Figure 17b, the UAV and nozzle controller receive the control instructions from the ground station and implement the position offset and nozzle flow adjustment.

(22) $$error=\frac{|V_{pred}-V_{expri}|}{V_{expri}}\times 100\%.$$

In accordance with the results of data comparison, the system can realize effective spray, which is reflected in the following aspects.

- The system can spray variably in accordance with the needs of farmland. As shown in Figure 17a,b, the deposition volume increases gradually from left to right and changes synchronously with the preset prescription value.
- The system can spray uniformly in accordance with the shape of farmland. As shown in Figure 17b, the difference between adjacent sample points is small in the central part of the area. Few sample points are found where spray is repeated or missed.
- The system can spray stably in accordance with the state of farmland. As shown in Table 5 and Figure 17b, the deposition volume fluctuation is weak with the violent change in state (flight, environment, nozzle).
- The system can spray visually in accordance with the log of ground station. As shown in Figure 17c, the difference in deposition volume between the prediction and experiment is small, and the errors at most sample points are below 30%.

However, gaps are found between the predicted deposition volume and the experimental deposition volume in the following aspects.

- The predicted deposition volume changes smoothly in the central part of the area, whereas the experimental deposition volume fluctuates. This condition is possibly due to the effect of different terrains and coverings on droplet movement, which is set to flat in the simulation.
- The experimental deposition volume changes gently at the boundary of the area, whereas the predicted deposition volume changes rapidly. This condition is possibly because the nozzle and the UAV take time to process the adjustment under real conditions, which is ignored in the prediction.
- The error is large in the margin part of the area. This condition is possibly because the simulation data are not in line with reality in the margin part of the area or the neural network is insensitive to the margin value.
- The error is small when the UAV enters the area, whereas the error is large when the UAV flies out of the area. This condition is possibly because the spray state is fixed over a period of time artificially and can not update in real time with the state data.

## 6. Conclusions and Future Work

In this study, a variable spray system with adjustable nozzle flow and controllable UAV offset is designed, discussed, and verified. A regression model is trained with the simulation data set verified by experiments to achieve uniform spray. This model is used to predict the distribution characteristics of droplet deposition in different spray states. The controllers offset the UAV position and adjust the nozzle flow. The conclusions of this research are as follows.

1. Field experiments show that CFD simulation can be used to predict the wind field and deposition distribution. The difference in downwash wind field is less than 1 m/s in most sample points. The errors of droplet deposition ratio are less than 10% in all sample points.
2. A simulation data set with 12 parameters is constructed. The 12 parameters are flight state (propeller speed, pitch angle, flight height), environment state (wind velocity, environment temperature, environment humidity), nozzle state (nozzle flow, droplet diameter, propeller nozzle distance), X−direction and Z−direction coordinates of sample points distributed in the range of 3 × 3 m, and droplet deposition ratio at the sample points.
3. An optimal regression model with five hidden layers (991, 833, 794, 767, 761) is selected in SVR and BPNN. SVR has low computation and requires few parameters to be set manually. However, the error of BPNN is 6.541%, indicating that BPNN is suitable for fitting the simulation data set.
4. The variable spray system achieves uniform deposition. In the farmland experiment, the deposition volume changes synchronously with the preset prescription value. The error of deposition volume between the prediction and experiment is within 30% in the central part of the area.

In the future, the quality and quantity of different spray states should be first improved in the simulation and used to produce perfect simulation data sets. Complex neural networks, such as long short-term memory, can be used to predict the distribution characteristics of droplet deposition.

## Figures and Tables

**Table 1:** Related work on the effect factors of spray droplet deposition.

Effect Factors Related Works	Propeller Speed	Pitch Angle	Flight Height	Wind Velocity	Env. Temperature	Env. Humidity	Nozzle Flow	Droplet Diameter	Nozzle Position
Chen et al. [18]				*√*				*√*	
Ahmad et al. [19]			*√*	*√*				*√*	
Wang et al. [20]	*√*			*√*	*√*			*√*	
Lv et al. [21]				*√*				*√*	
Zhu et al. [22]		*√*	*√*	*√*			*√*		
Ling et al. [23]	*√*	*√*	*√*	*√*				*√*	
Richardson et al. [24]	*√*			*√*				*√*	
Richardson et al. [25]			*√*	*√*				*√*	*√*
Qi et al. [26]					*√*	*√*			
Chen et al. [27]	*√*		*√*	*√*					*√*
Liao et al. [28]				*√*				*√*	

**Table 2:** Parameter setting of UAV in CFD simulation.

Name	Region	Region	Region	Cells	Maximum	Boundary
	Shape	Number	Specification	Number	Skewness	Condition
static region	cylinder	1	radius is 4500 mm, height is 3000 mm	351,660	about 0.94	top and side is velocity inlet, bottom is wall
dynamic region	cylinder	8	radius is 276 mm, height is 300 mm	about 1,800,000	about 0.79	interface
empty region (propeller)	propeller	8	diameter is 533 mm, pitch is 178 mm	null	null	wall
empty region (control module)	cube	1	240 mm × 240 mm × 80 mm	null	null	wall
empty region (water tank)	cube	1	360 mm × 360 mm × 200 mm	null	null	wall

**Table 3:** Variation range of nine factors in orthogonal simulation.

Effect Factor	Minimum Value	Maximum Value	Interval
propeller speed (rpm)	3000	5000	500
pitch angle (${}^{\circ }$)	0	20	5
flight height (m)	1	4	1
wind velocity (m/s)	0	2	0.5
environment temperature (${}^{\circ }$C)	10	40	10
environment humidity (%)	50	100	25
nozzle flow (L/min)	0.1	0.5	0.1
droplet diameter (μm)	150	250	50
propeller nozzle distance (cm)	20	40	10

**Table 4:** Performance of regression models with different parameters on deposition ratio simulation data set.

Model	Parameter	RMSE-Train (%)	RMSE-Test (%)	MAE-Train (%)	MAE-Test (%)	R^2^-Test
SVR	linear	11.240	11.087	9.929	9.961	0.763
SVR	poly (degree = 2)	11.203	11.049	9.887	9.919	0.779
SVR	poly (degree = 3)	11.232	11.080	9.920	9.954	0.772
SVR	RBF	11.006	10.835	9.673	9.684	0.816
SVR	sigmoid	10.613	10.536	8,974	9.045	0.813
BPNN	2 (2599, 2593)	7.199	7.795	3.549	3.892	0.836
BPNN	3 (1745, 1733, 1715)	5.662	6.981	2.369	2.776	0.864
BPNN	4 (1031, 977, 905, 803)	5.776	6.789	2.457	2.904	0.876
BPNN	5 (991, 833, 794, 767, 761)	5.265	6.541	2.054	2.532	0.873
BPNN	6 (651, 645, 616, 573, 519, 501)	5.731	6.784	2.486	2.719	0.885
BPNN	7 (409, 397, 373, 361, 343, 325, 313)	5.640	6.927	2.254	2.659	0.865

**Table 5:** State data sent to the server, and the center offset distance and effective deposition radius calculated in accordance with the data are returned from the server (a quarter of all data).

Number	Propeller Speed	Pitch Angle	Flight Height	Wind Velocity	Env. Temperature	Env. Humidity	Droplet Diameter	Prop-Nozz Distance	Nozzle Flow	UAV Offset
Unit	(rpm)	(${}^{\circ }$)	(m)	(m/s)	(${}^{\circ }$C)	(%)	(μm)	(cm)	(L/min)	(m)
1	4323	3	2.5	1.1	17.92	92	141	0.3	0.326	1.7
2	4260	3	2.2	0.7	17.63	93	160	0.3	0.371	1.1
3	4130	3	1.9	0.9	17.75	92	144	0.3	0.291	1.5
4	4077	3	2.1	0.4	18.06	94	143	0.3	0.337	0.5
5	3950	3	2.0	0.4	17.59	95	150	0.3	0.270	0.5
6	3860	3	2.4	0.7	17.95	92	160	0.3	0.248	1.2
7	3814	3	2.0	1.0	18.04	92	206	0.3	0.169	1.6
8	3752	2	1.7	0.4	18.07	91	185	0.3	0.203	0.5
9	3700	2	1.9	0.2	17.84	87	179	0.3	0.213	0.3
10	3663	2	2.3	0.4	17.93	88	225	0.3	0.135	0.5
11	3628	2	2.1	0.1	17.69	88	249	0.3	0.073	0.1
12	3573	2	1.9	0.0	18.13	87	249	0.3	0.075	0.0

## Data Availability

The data used to support the findings of this study are available from the author (H.W.) upon request.

## Abbreviations

The following abbreviations are used in this manuscript:

UAV	Unmanned aerial vehicle
PA	Precision agriculture
CFD	Computational fluid dynamics
VMD	Volume median diameter
Env.	Environment
FVM	Finite volume method
LBM	Lattice Boltzmann method
PLSR	Partial least squares regression
SVM	Support vector machine
DSS	Decision support system
ANN	Artificial neural network
DPM	Discrete phase model
SST	Shear-stress transport
PET	Polyethylene terephthalate
SVR	Support vector regression
BPNN	Back propagation neural network
KKT	Karush-Kuhn-Tucker
RMSE	Root mean square error
MAE	Mean absolute error
$R^{2}$	Coefficient of determination
